# Immunohistopathology of Cochleovestibular Schwannoma in Human Temporal Bone Specimens

**DOI:** 10.3390/biology14111540

**Published:** 2025-11-03

**Authors:** Jennifer T. O’Malley, Anat O. Stemmer-Rachamimov, Sebahattin Cureoglu, Michael J. McKenna, D. Bradley Welling, Alicia M. Quesnel

**Affiliations:** 1 Otopathology Laboratory, Massachusetts Eye and Ear, Boston, MA 02114, USA; 2 Department of Neuropathology, Massachusetts General Hospital and Harvard Medical School, Boston, MA 02114, USA; astemmerrachamimov@mgh.harvard.edu; 3 Otopathology Laboratory, Department of Otolaryngology-Head & Neck Surgery, University of Minnesota, Minneapolis, MN 55455, USA; cureo003@umn.edu; 4 Akouos, Inc., a wholly owned subsidiary of Eli Lilly, Boston, MA 02210, USA; michael.mckenna@lilly.com; 5 Department of Otolaryngology—Head and Neck Surgery, Harvard Medical School, Boston, MA 02114, USA; brad_welling@meei.harvard.edu (D.B.W.); alicia_quesnel@meei.harvard.edu (A.M.Q.); 6 Otopathology Laboratory, Massachusetts Eye and Ear, Department of Otolaryngology—Head and Neck Surgery, Harvard Medical School, Boston, MA 02114, USA

**Keywords:** cochlear, schwannoma, myelin, macrophages, tumorlets, inflammation

## Abstract

**Simple Summary:**

This qualitative study looked at how cochleovestibular schwannomas—noncancerous tumors that grow on the hearing and balance nerves—may cause hearing loss. While hearing tests like the auditory brainstem response (ABR) suggest damage to the hearing nerve, the exact cause has not been well understood. Using 28 tumor samples from human temporal bones, we examined how these tumors interact with the hearing nerve. We found that the tumor sometimes pushes the nerve fibers aside or grows in between them. Inside all tumors, we identified nerve fibers, some of which were bent in abnormal directions. We also found signs of nerve degeneration, including damaged myelin (the protective coating around nerves) and structures called “onion bulbs” that may mark early nerve breakdown. In many cases, there was no clear boundary between the tumor and nerve tissue—they were mixed and sometimes involved more than one nerve. We also observed signs of inflammation, including immune cells called macrophages. These findings suggest hearing loss is caused not just by tumor pressure, but also by damage to cochlear nerve fibers, their coating, and early inflammation. Tumor growth may occur by spreading between healthy nerve fibers rather than simply pushing them aside.

**Abstract:**

The aim of this study was to investigate the pathology of hearing loss caused by cochleo-vestibular schwannoma. Surgical specimens have demonstrated that a tumor may displace normal nerve fibers of the cochlear nerve to one side (pushing pattern) or the neoplastic cells may invade the tumor and grow between normal nerve fibers (infiltrating pattern). The goal was to study the relationship of the tumor to the remaining fibers of the cochlear nerve. Nerve fibers within all 28 tumors showed positive anti-neurofilament (NF) labeling. Axons within tumors were sometimes turned orthogonal to their original plane. Onion bulb formations were observed in tumors giving rise to early Antoni B-like regions of degeneration. Positive anti-myelin protein zero (MPZ) labeling was demonstrated. No clear capsule was found between tumor and nerve. There was a comingling of tumor and nerve fibers either with the nerve of origin or with both the nerve of origin and surrounding internal auditory canal nerves. Iba1+ macrophages were prevalent within cochleovestibular schwannomas. Our results suggest that retro cochlear mechanisms of hearing loss go beyond compression of the eighth cranial nerve, involve both myelin and axon degeneration, and suggest an inflammatory component from the earliest stage of the disease.

## 1. Introduction

Cochleovestibular schwannomas (CVS, including vestibular schwannomas formerly termed acoustic neuromas [1]) make up roughly 85% of all cerebellopontine angle tumors [2,3]. These tumors arise from the Schwann cells of the vestibular and sometimes cochlear nerve in a 3.2:1 ratio [1]. They occur in sporadic (unilateral) and familial (bilateral) forms. Sporadic CVS are caused by somatic mutation within vestibulocochlear nerve Schwann cells [4], whereas NF2-related schwannomatosis, formerly neurofibromatosis type 2 or NF2 [5,6], is caused by systemic mutation in the NF2 gene on 22q12.2 [4,7,8]. It is necessary for two somatic mutations to occur in a single cell to escape the normal function of the NF2 gene which acts as a tumor suppressor [9]. In NF2-related schwannomatosis, one mutation is inherited in an autosomal dominant fashion and the other mutation then occurs, often as a loss of heterozygosity. Sensorineural hearing loss (SNHL) resulting from these tumors has been thought to occur from both retro cochlear [10,11,12,13,14] and cochlear mechanisms [1,15,16,17,18,19,20,21]. Cochlear mechanisms may be due to direct tumor invasion of the cochlea or secondary to excreted ototoxic proteins from the tumor entering the cochlea. However, these mechanisms are not well understood. The etiology of hearing loss in patients with CVS remains unclear [22]. 

Both historically and currently, schwannomas are described as growing within a capsule that remains peripherally attached to the parent nerve [23,24,25,26]. They have been described as “encapsulated benign tumors almost entirely composed of Schwann cells that are perched on but not comingled with nerve bundles” [27]. Others have suggested that entrapped axons are not a common feature [24] of schwannomas. Their lack of anti-neurofilament staining within the body of the tumor has been used to differentially diagnose them from neurofibromas [28]. Schwannomas have been thought to damage nerve fibers by expanding within their capsules and compressing fibers while neurofibromas are believed to diffusely expand the involved nerves and encompass included axons [24]. The United States Department of Health and Human Services consensus statement on acoustic neuroma in 1991 stated, “these tumors are encapsulated, round, and usually appear as a single mass.” The statement went on to suggest that the tumors in NF2 (bilateral familial type) can infiltrate the fibers of individual nerves [29]. This concept that embedded axons of the eighth nerve are seen in histopathology of NF2 tumors but rarely seen in unilateral sporadic tumors has been repeated by others since 1991 [9,30]. Contrary to this, there are reports from surgical specimens showing that as one of these tumors arise, it usually only invades its nerve of origin and as it grows other nerves, including the cochlear nerve, can become microscopically invaded by tumor cells [31]. Nerve fibers become surrounded by tumor cells in sporadic cases and the tumor–nerve interface is gradual and not abrupt [32]. These findings were corroborated in a study by Neely [33] and again by Neely and Hough [34,35] in two very small intracanalicular solitary schwannomas. Neely and Hough [35] made a point of mentioning that contrary to their experience with larger tumors, “the incorporated nerve fiber population within the substance of the tumor mass was quite abundant” in the two very small tumors. Similar results of cochlear nerve involvement in the tumoral process as evidenced by no clear cleavage plane between nerve and tumor were reported in 9 of 12 surgical specimens of acoustic neurinomas [36]. An immunohistochemical study of 10 intact medium sized surgically removed CVS with antibodies against human neurofilaments [37] showed tumoral invasion of the cochlear nerve in 6 out of 10 specimens.

Taken together, these reports suggest that both sporadic schwannomas and NF2 tumors can infiltrate both the nerve of origin and other nerves in proximity to the tumor within the internal auditory canal (IAC). Schwannoma tumor cells have direct access to the nerve fibers as there is neither a clearly defined cleavage plane nor capsule around many of these tumors. As these tumors grow and expand, it is reasonable to expect that there would be both infiltration and pushing effects exerted upon the eighth nerve. The pattern of involvement has important surgical implications: a pushing pattern is far more amenable to successful hearing preservation than an infiltrative one. Furthermore, the pattern of involvement may provide clues to tumorigenesis itself.

There is renewed and emerging interest in the role of the tumor microenvironment in CVS [22,38,39]. The tumor microenvironment includes the tumor nerve interface. A very recent review stated that, “it is incumbent upon researchers to advance our understanding of this area” [38]. Surgery is often difficult due to loss in section planes because these tumors are extremely adherent to the cochleovestibular nerves [40]. Furthermore, such adhesions pose significant risk to the cochleovestibular nerves during dissection [40] which could lead to total deafness. The consistency of a CVS, or its biomechanical stiffness, is associated with worse clinical outcomes in both benign and malignant tumors [41]. Stiffer CVS are less amenable to surgery with increased risk of injury at the tumor–nerve interface [41]. However, reasons why some tumors can be stiffer than others are not completely known. It has been suggested that how these tumors originate, are sustained, and grow, can be better understood by studying the surroundings of the tumor more carefully [39]. Nickl et al., 2024, further purported that inflammation and the tumor microenvironment may play a role in tumor formation and nerve infiltration [39]. Being able to better identify the cellular components of the tumor microenvironment, including its interaction with surrounding nerve fibers, is crucial to our knowledge base of CVS. New information regarding the substance of the tumor and its surroundings is extremely important to the development of new therapies to improve patient care [38].

In conventionally prepared human temporal bones stained with hematoxylin and eosin, the pattern of involvement of the tumor and nerve is difficult to ascertain. Both tumor cells and normal nerve fibers take up the eosin stain. The present study involves systematic investigation of the pattern of involvement of both the cochlear and vestibular nerves using immunomarkers of normal nerve tissue. Staining for neural and myelin proteins in adjacent sections permits addressing the issue of whether there are selective adverse effects of the tumor upon the different elements of the nerve. Utilizing temporal bone specimens rather than surgical specimens affords the opportunity to investigate the relationship of the tumor to the remaining fibers of the VIIIth nerve. It provides us with an expansive view to gain further insight into the tumor microenvironment and positions this study in direct dialog with recent advances and currently debated topics of possible mechanisms contributing to hearing loss associated with CVS.

## 2. Materials and Methods

The archival temporal bone collections at the Massachusetts Eye and Ear (MEE) and the University of Minnesota were searched, utilizing the National Temporal Bone Registry database where that data was maintained, to find unilateral untreated cochleovestibular schwannoma. A total of 26 cases were identified. Both symptomatic (auditory or vestibular complaints) and occult (found on temporal bone histopathology post-mortem) cases were included. Two cases of unresected neurofibromatosis type 2 (NF2) were also included. All 28 of the specimens were conventionally processed for light microscopy. The temporal bones were fixed in formalin, decalcified in either trichloroacetic acid or ethylenediaminetetracetate. They were embedded in celloidin, serially sectioned at 20 µm in the axial plane, and every 10th section was stained using hematoxylin and eosin (H&E).

The tumors were observed in the H&E celloidin sections using a Nikon E800 microscope (Nikon Inc., Melville, NY, USA). Six sections (two serial sections each from the superior, middle, and inferior portions of the tumors) were chosen for immunolabeling with mouse anti-neurofilament, 200 kD (Boehringer Mannheim, Mannheim, Germany) at a dilution of 1:2000 and chicken anti-myelin protein zero (Abcam, Cambridge, MA, USA) antibodies. Three sections were labeled for neurofilament and three for MPZ using a protocol that is described in detail in previous publications [42]. The method involved mounting the sections on gelatin subbed slides and removing the celloidin with a mixture of methanol and sodium hydroxide. Labeling with primary and secondary antibodies was followed by application of avidin-biotin-horseradish peroxidase, Standard ABC Kit (Vector Labs, Burlingame, CA, USA) and colorization with diaminobenzidine (DAB) and hydrogen peroxide. Finally, Permount (Thermo Fisher Scientific, Waltham, MA, USA) was applied and slides were cover slipped. Slides were reviewed and photographed using a Nikon E800 microscope. High-power images were taken with a 100× oil-immersion objective (NA = 1.3) and differential interference optics.

The immunolabeling patterns were classified as pushing (tumor cells push normal nerve fibers to one side) or infiltrative (tumor cells infiltrate between normal nerve fibers). Each of the sections was scored as +/− for presence/absence of immunolabeled structures. Data was gathered regarding tumor type, tumor size, nerve of origin, Antoni subtype, and, if available, ABR and speech audiometry data.

When it was observed that there was an inflammatory response in the tumors, an additional immunomarker for macrophages was applied to sections close to those that had been labeled with the neuronal and myelin markers on a subset of the specimens (13/19 of the MEE specimens for which extra sections were readily available). The macrophage marker was a rabbit primary antibody against ionized calcium binding adaptor molecule 1 (Iba1) (Wako chemicals USA, Inc., Richmond, VA, USA) at a dilution of 1:2000. Conventional DAB immunostaining was caried out as stated previously.

Immunofluorescence labeling was completed to multiplex the NF200, MPZ, and Iba1 together in four cases (ID#s 1, 2, 3, and 6). For that protocol, sections were placed in a blocking buffer (phosphate-buffered saline with 5% normal horse serum and 0.3–1% Triton X-100 for 1 h at room temperature) followed by an overnight incubation at room temperature in the primary cocktail containing mouse anti-NF200 at 1:1000, chicken anti-myelin protein zero at 1:100, and rabbit anti-Iba1 at 1:100. Three rinses in buffer followed the next morning. Primary incubations were followed by 2 sequential 60 min incubations at room temperature in species-appropriate secondary antibodies (coupled to Alexafluor dyes) with 0.3–1% Triton X. After immunostaining, Vectashield (Vector Labs, Burlingame, CA, USA) was applied followed by coverslips. Coverslips were sealed with nail polish. Images were acquired on a Leica SP8 confocal microscope.

A detailed methodology workflow can be found in Supplementary Materials.

## 3. Results

The 28 specimens studied here came from 9 female and 19 male temporal bone donors (Table 1). The ages ranged from 43 to 100 years. A total of 26 out of the 28 showed both infiltrative and pushing patterns of involvement, while 2 showed only infiltrative (very small intralabyrinthine, ID#s 16 and 17, Table 1). The two intralabyrinthine tumors demonstrated early Antoni B-type histology. There were areas of early degeneration with no areas of palisading. The remaining 26 tumors (Table 1) contained areas of both Antoni A and B regions. Tumor diameters ranged from 0.3 mm to 15 mm (two were too extensive to measure reliably). Of the 19 specimens from MEE for which there was more complete data, 15 of the tumors were occult and 4 were clinically diagnosed. There was only ABR data for 2 out of the 28 cases. For both cases (ID#2 and #12), ABR results were thought to be diagnostic of retro cochlear lesions with both showing prolonged latencies in waves I-III compared to the contralateral non-tumor ear (Table 1).

**Table 2:** CVS-nerve of origin and cochlear invasion (check means yes).

ID #	Nerve of Origin	Cochlear Nerve Invasion
1	Too large to determine	✓
2	Too large to determine (both divisions of vestibular and cochlear)	✓
3	Inferior vestibular and cochlear	✓
4	Superior division of vestibular	✓
5	Too large to determine (both divisions of vestibular and cochlear)	✓
6	Vestibular	✓
7	Cochlear	✓
8	Inferior division of vestibular	✓
9	Superior division of vestibular	no
10	Inferior division of vestibular	no
11	Superior division of vestibular	no
12	Too large to determine	✓
13	Intralabyrinthine to vestibular	✓
14	Cochlear	✓
15	Vestibular	no
16	Cochlear—intralabyrinthine	✓
17	Cochlear—intralabyrinthine	✓
18	Too large to determine (auditory, vestibular, and facial)	✓
19	Superior vestibular	no
20	Inferior vestibular	✓
21	Superior vestibular	✓
22	Superior vestibular	no
23	Superior vestibular	✓
24	Superior vestibular	no
25	Cochlear	✓
26	Superior vestibular	✓
27	Superior vestibular	✓
28	Too large to determine (both auditory and facial)	✓

This study comprised 168 immunolabeled sections through cochleovestibular schwannomas. All 28 specimens showed positive labeling for NF or myelin within the tumors (Table 3). Overall, 83% of the slides which had been labeled for anti-NF (70 out of 84) were positive and 77% of the slides labeled for anti-MPZ (65 out of 84) were positive (Table 3). Overall, 64% of the anti-NF-stained sections showed evidence of Wallerian degeneration characterized by neuronal swelling, blebbing, and axonal ovoids (Table 3).

**Figure 1:**
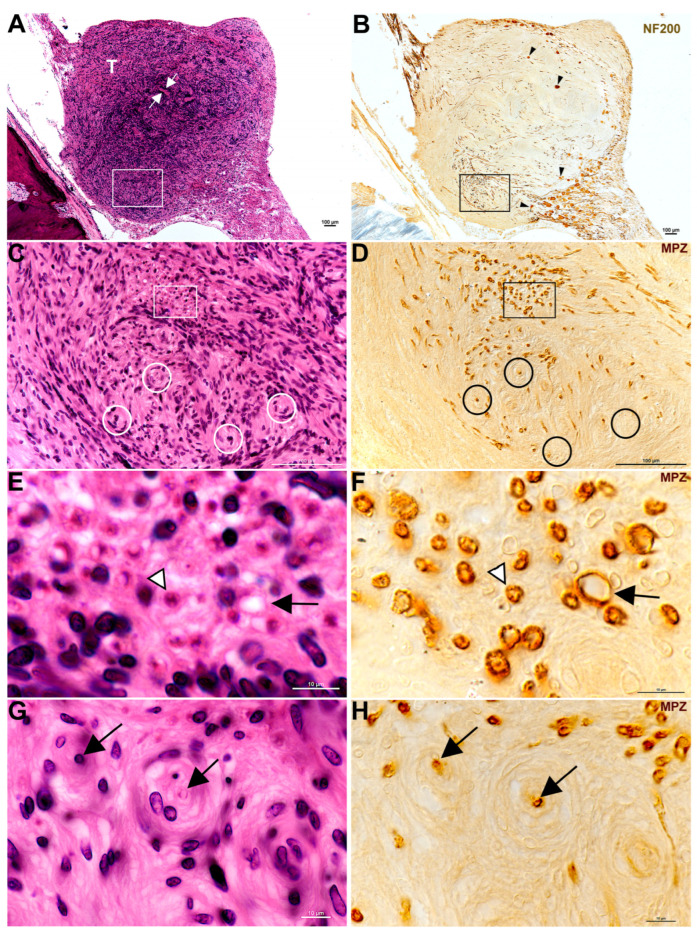
Shows a tumor and nerve from ID #15 (Table 1), a 91-year-old female. Panels (**A**,**C**,**E**,**G**) show a single hematoxylin and eosin-stained section. Panels B, D, F, and H show a near-serial section to those shown on the left-hand side that is stained for the NF200 Panel (**B**) and MPZ Panels (**D**,**F**,**H**). Both sets of vertical panels are set up with similar viewing areas for easy comparison. Panel (**A**) shows the tumor (T) and white arrows pointing to one Antoni A area in the tumor. The white boxed area outlines the area shown in Panel (**C**). Panel (**B**) shows the tumor (T) labeled with NF200. Nerve fibers are evident, both within the tumor and along the axons extending both peripherally and centrally. Scarpa’s ganglion cells (black arrowheads) are also evident within the body of the tumor. The black box outlines Panel (**F**). In Panel (**C**), onion bulb formations (white circles) are apparent as circles with a center that is sometimes empty or occupied. The white box in Panel (**C**) outlines Panels (**E**). Panel (**D**) (MPZ label) reveals that what is inside of the onion bulb (black circles) is sometimes a nerve fiber in a plane orthogonal to its original course. The two black circles further to the left in Panel (**D**) each show a myelinated nerve fiber cut in cross section. The other two black circles in Panel (**D**), lower center and right of center, are around onion bulbs with no apparent fiber visible at their center, presumably demonstrating that the nerve fibers are already demyelinated or fully degenerated. The black box in Panel (**D**) demarcates the contents of Panel (**F**). Panel (**E**) appears to show a bundle of nerve fibers that are running orthogonally to the original plane. The black arrow may be an early onion bulb forming. The white and black arrowhead is pointing to an eosinophilic density. When stained with an antibody against MPZ, it is revealed that the density is a myelinated nerve fiber cut in cross section, shown by the black and white arrowhead on Panel (**F**). Panel (**F**) also shows what might be considered an early onion bulb forming. It too has, at its center, a myelinated nerve fiber in cross section (black arrow in Panel (**F**)), like the fully formed onion. Panel (**G**) shows two onion bulbs at higher magnification (black arrows) labeled with anti-MPZ that are also seen in Panel (**H**) (black arrows). The onion bulb areas may become early Antoni B areas as spaces appear to develop surrounding these orthogonal fibers. Please note these white spaces within the onion bulbs. Figure 1 captures the lack of a capsule around the tumor as ganglion cells appear within the tumor matrix. One can also track nerve bundles all the way through the tumor. Figure 1 also demonstrates fiber disarray, onion bulb formation, and presumably demyelination, as some onion bulbs appear to be missing myelin at their centers.

**Figure 2:**
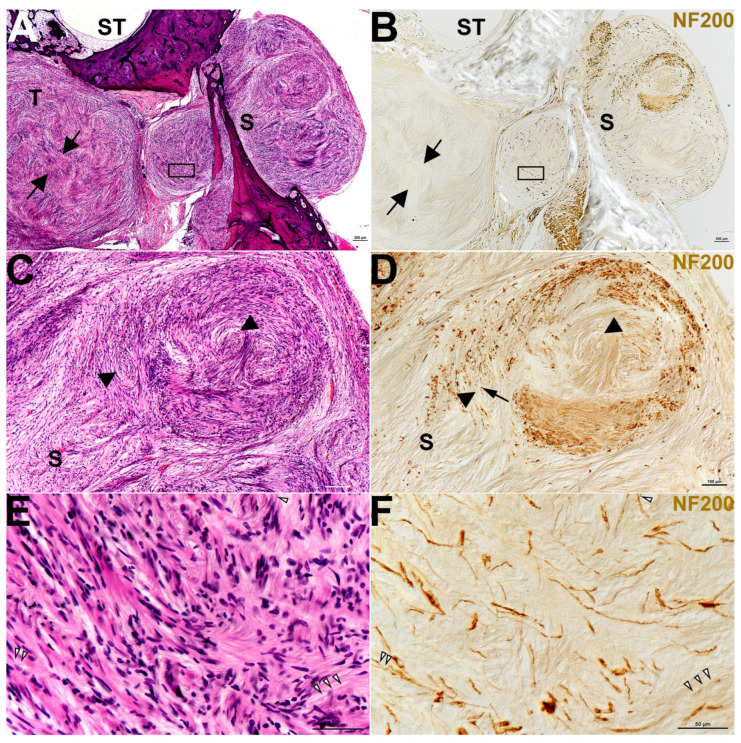
Demonstrates the nerve and tumor from ID #12, right ear (Table 1) from a 43-year-old female with NF2-related schwannomatosis. Panels (**A**,**C**,**E**) show a single hematoxylin and eosin-stained section. Panels (**B**,**D**,**F**) show a section, serial to the previous section, stained with an anti-NF200 antibody and a conventional DAB reaction product in brown. The areas from the left-hand side and right-hand side are set up as matching pairs for easy comparison. Panel (**A**) includes part of the scala tympani (ST) of the cochlea and the area that was once the saccule (S) but now is occupied by tumor (T). The black arrows point to one Antoni A-type area of the tumor. The black box demarcates Panel (**E**). In Panel (**B**), the NF200 label is striking around the saccule (S), where a large swirl of nerve fibers appears to have been pushed through the saccule (S) with the tumor tissue. There is no identifiable saccular tissue remaining, only tumor and nerve fibers. The black box demarcates Panel (**F**). Panels (**C**,**D**) show the saccular (S) area in higher magnification. Black arrowheads, in panels (**C**,**D**), point toward some of the Antoni B-type areas in the tumor. The black arrow in (**D**) is pointing toward a NF200-positive nerve fiber cut in cross section, which is orthogonal to its original plane. Panels (**E**,**F**) (both demarcated by black boxes in Panels (**A**,**B**)), are a mix of tumor and nerve fiber. The NF200 antibody label (in Panel (**F**)) is necessary to understand exactly where the nerve fibers remain within the tumor. There are several thin pink lines that might be disorganized nerve fibers in Panel (**E**) (white and black arrowheads), but having the NF200 label in Panel (**F**) (white and black arrowheads to match Panel (**E**)) makes fiber identification possible.

**Figure 3:**
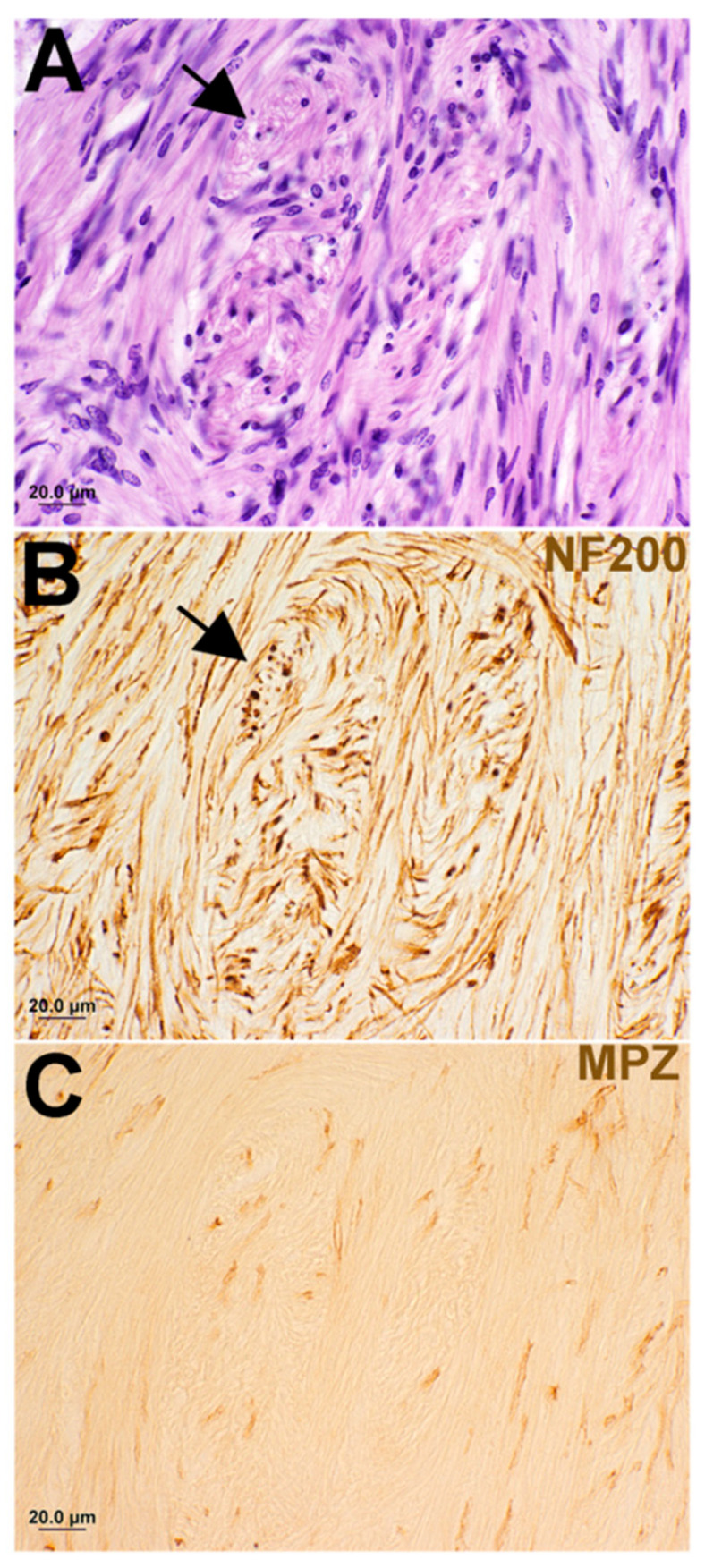
Shows the same individual as in Figure 2, but from a distant area of the tumor. Panels (**A**–**C**) are only 20 µm apart from each other and so are serial sections. Panel (**A**) is the hematoxylin and eosin-stained section, Panel (**B**) is labeled with anti-NF200, and Panel (**C**) is labeled with anti-MPZ. The black arrows in both (**A**,**B**) point to onion bulbs or early Antoni B areas of the tumor. What is obvious in these three panels is that it is difficult to understand from the hematoxylin and eosin sections just how many nerve fibers remain in an area like this. There are many disorganized nerve fibers within the body of the tumor. What is also evident is that there is markedly less MPZ label (Panel (**C**)) than there is NF200 label (Panel (**B**)). It is as if the tumor destroys the myelin prior to the NF200. The fibers seem to be dying from the outside inwards.

**Figure 4:**
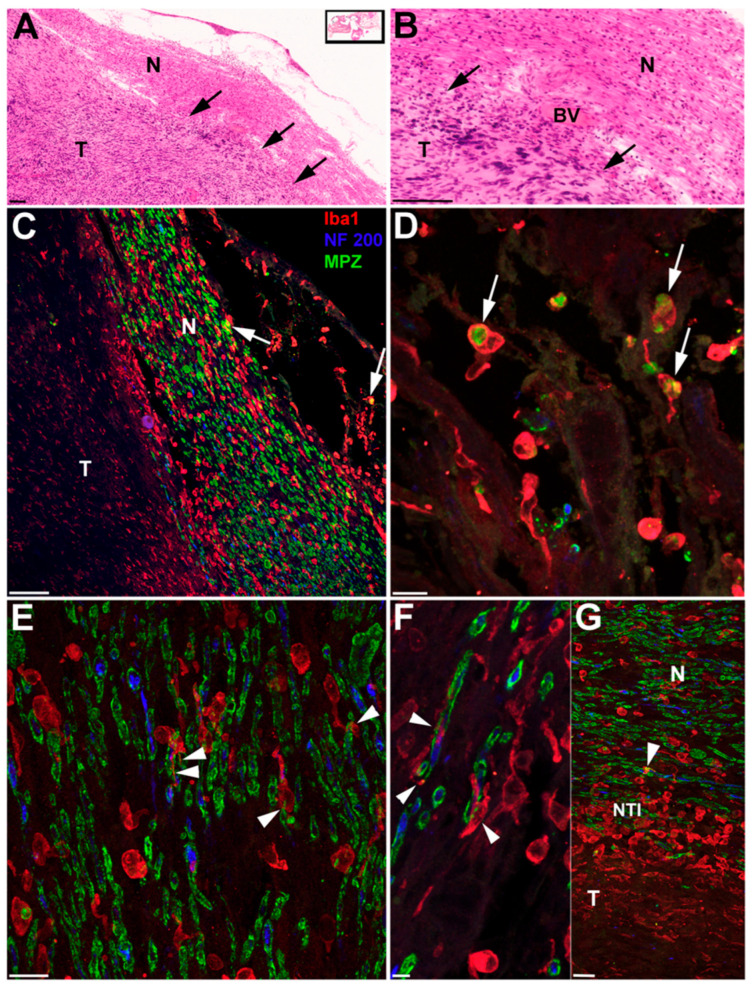
Demonstrates the nerve and tumor with accompanying macrophages from a 100-year-old female who had 0% discrimination in this ear. All panels show two sections from ID #6 (Table 1). Panels (**A**,**B**) are from a single hematoxylin and eosin-stained celloidin section (boxed inset Panel (**A**)) and panels (**C**–**G**) are from a second celloidin section, approximately 100 µm from the first, with immunofluorescence labeling for Iba1 (macrophages in red), NF200 (nerve fibers in blue), and MPZ (myelin in green). Panel (**A**) shows the cochlear nerve (N) and tumor (T). The black arrows are pointing toward the nerve–tumor interface. Panel (**B**) is a higher magnification of the nerve–tumor interface with a blood vessel (BV) in view. Calibration bars in both (**A**) and (**B**) are equal to 100um. Panel (**C**) is from the same general area as Panel (**A**), only tilted clockwise by approximately 35 degrees. Once again, the nerve (N) and tumor (T) are shown. The red labeled cells are positive for Iba1, denoting macrophages. The blue label for NF200 can be seen within nerve fibers. And the green label shows the myelin surrounding the nerve fibers. At the tips of both white arrows, are macrophages filled with myelin (greenish yellow inside the red circles). Panel (**D**)’s white arrows demonstrate a higher magnification of macrophages filled with myelin (green within the red border of the macrophage cell membranes). Panel (**E**) demonstrates thin macrophage processes (white arrowheads) surrounding nerve fibers (green around the blue profiles) in what may be the initial step to engulfing the fiber completely. In Panel (**F**), the white arrowheads point to further engulfment, where macrophage cytoplasm is seen penetrating the axon (red coming out of frame through the blue at center of nerve fiber (topmost white arrowhead), while the lower left side white arrowhead shows a process of the macrophage holding on to the fiber. The white arrowhead, in the center of the frame, shows a similar process occurring to a different nerve fiber. Panel (**G**) shows nerve (N), tumor (T), and the nerve–tumor interface (NTI). The white arrowhead points again toward a macrophage (red) wrapped around the outside of a nerve fiber (green myelin around blue neurofilament). Please note that from the top of Panel (**G**) toward the bottom of Panel (**G**) there is a shape change that occurs in the macrophages (cells in red). At the nerve–tumor interface (NTI) this is most obvious, where large round amoeboid macrophages (red) are seen in great abundance. Elsewhere macrophages (red) are seemingly more ramified, with long thinner processes. It would appear from this image that macrophage activation is occurring at the nerve–tumor interface. Calibration lines in Panels (**C**–**G**) are equal to 10 µm.

**Figure 5:**
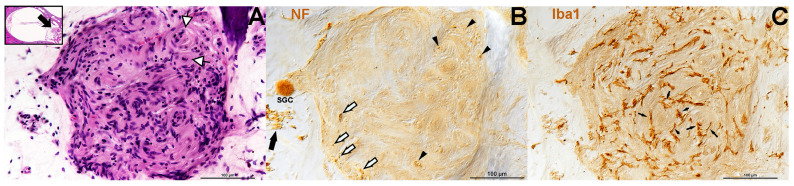
Shown here is the ear from ID#16 (Table 1), an 82-year-old male with a tiny (tumorlet) occult schwannoma within Rosenthal’s canal. The hematoxylin and eosin-stained section is seen in Panel (**A**). White and black arrows are pointing to onion bulb formations. In Panel (**B**), a serial section to Panel (**A**) has been stained with an antibody against NF to label nerve fibers. The filled black arrowheads at the top right and bottom center point to nerve fibers at the center of onion bulbs, within the tumor. The black and white arrows, lower left of center, show nerve fibers within the tumor mass. The single large black arrow, far left, shows nerve fibers near the tumor. A single spiral ganglion cell (SGC) is positive for NF. In Panel (**C**), a serial section to Panels (**A**,**B**) has been stained with an antibody against Iba1 to label macrophages. The black arrows are pointing out that the macrophages are sometimes inserting their processes into the onion bulbs (lower right). Other onion bulbs are circled by what seems like many macrophages at once (black arrows more at center of image (**B**)). Overall, Panel (**B**) reveals many Iba1-positive macrophages throughout this tumorlet.

Findings from this study exhibited plentiful nerve fibers within CVS. All specimens analyzed contained either anti-NF labeling or anti-MPZ labeling or both to some extent (Table 3 and Figure 1, Figure 2, Figure 3, Figure 4 and Figure 5). Some of the tumors demonstrated areas with few fibers (Figure 1B, Figure 2B, and Figure 4C), while other areas were filled (Figure 1D,F, Figure 2D, Figure 3B and Figure 4C). Fiber disarray/disorganization (Figure 1B,D,F,H, Figure 2B,D,F and Figure 3B,C) was present in the small tumors and the large tumors. There was no clear capsule or membrane surrounding the tumors. In most instances, tumor and nerve fibers comingled (Figure 1B, Figure 2B,D,F, Figure 3B and Figure 4A,B,C,G). Ganglion cells were displaced within the tumor (Figure 1B). Axons sometimes appeared orthogonal to the original plane (Figure 1B,D,F,H, Figure 2B,D and Figure 3B).

Degenerating nerves were also observed. Demyelination [Figure 1D,F,H, Figure 3C and Figure 4C–G] did not appear related to tumor size or solely from compression of fibers. Demyelination was observed in some of the smallest tumors where there was no compression of fibers. Degenerative areas with onion bulbs appeared as early Antoni B-type regions (Figure 1C–H, Figure 3A–C, and Figure 5A,B).

A plane between tumor and nerve was not obvious (Figure 1, Figure 2, Figure 3 and Figure 4). Both axons (Figure 1, Figure 2, Figure 3 and Figure 4) and Scarpa’s ganglion cells (Figure 1B) could be seen within the body of these tumors on occasion. Even the smallest of these tumors, known as tumorlets, contained numerous nerve fibers. Cochleovestibular schwannoma cases studied here demonstrated neurofilament labeling within the body of the tumor and sometimes at the tumor margins. There was anti-MPZ labeling present in the body of these tumors as well (Figure 1D,F,H, Figure 3C, Figure 4C–G). It was not possible to quantify the number of fibers. Often there were areas of tumors that appeared as if the myelin had degenerated prior to the neurofilament, as if the fibers were dying from the outside in (Figure 3).

Previous work has shown that macrophages were present along the eighth nerve in normal aging ears [43]. Additionally, it appeared that many of the tumors had an inflammatory component. In 13 of the 19 MEE specimens for which extra sections were readily available, labeling for Iba1 revealed plentiful macrophages in all tumors that were stained. Macrophages were present in both the largest tumors [Figure 4] and in the smallest tumors [Figure 5]. Macrophages appeared in both Antoni A areas and Antoni B areas of tumors [Figure 4 and Figure 5]. Morphologically, the macrophages demonstrated at least two types, ramified and amoeboid [Figure 4C–G and Figure 5B]. Some of the macrophages appeared to be in a transitional state demonstrating both a rounded cytoplasmic area together with multiple cytoplasmic extensions or ramifications [Figure 4C–G]. Multiplexing the NF200 together with the MPZ and Iba1, revealed activated macrophages containing myelin [Figure 4D]. At the nerve–tumor interface (NTI), where tumor cells were actively invading and comingling with remaining “healthier looking” nerve fibers [Figure 4A–G], activated amoeboid macrophages were particularly prevalent [Figure 4G]. Also noted were partially activated (transitional) macrophages with processes surrounding nerve fibers and their myelin sheaths [Figure 4E,F]. Macrophage processes also appeared inserted into the axoplasm of nerve fibers [Figure 4F]. Macrophage processes were seen inserted within the layers of onion bulbs [Figure 5B].

## 4. Discussion

In the present study, we focused specifically on qualitatively documenting possible histopathologic correlations of retro cochlear mechanisms of hearing loss associated with cochleovestibular schwannomas. It was a reasonable assumption for many years that compression of the cochlear nerve in the internal auditory canal was one possible cause of retro cochlear hearing loss associated with cochleovestibular schwannomas. It is very probable that axons within the IAC can be compressed as these tumors grow. In addition to a compression mechanism, the findings in this report of demyelination of the nerve, degeneration of the axons, and presence of macrophages, also shown by others [44,45,46,47,48,49,50] in large numbers even within the smallest of these tumors (tumorlets) indicates that there are other probable mechanisms that can contribute to retro cochlear hearing loss. Beyond that, our finding in a previous report [43], that there are macrophages present in normal ears (with no known hearing loss other than from changes due to age) along the VIIIth nerve within the IAC and cochlea, indicates that the innate immune system is present jointly with healthy Schwann cells. So, prior to any tumorigenesis, macrophages are present in the local milieu. It is not known to what extent macrophages contribute to a neoplastic transformation. However, there is evidence that trauma [51,52,53], chronic inflammation [54], radiation [55], or infection [56] may contribute to carcinogenesis and tumor formation. Two cases are reported specific to the formation of schwannoma following repeated trauma to the sciatic nerve and tibial nerve [52,53]. Activation of macrophages is known to liberate inflammatory cytokines and chemokines involved in inflammation and its resolution. However, chronic activation can result in bystander damage. Activated inflammatory cells can be sources of reactive oxygen species (ROS) and reactive nitrogen intermediates, which in turn may induce DNA damage and genomic instability [54]. Cytokines such as TNF-α can also cause accumulation of ROS in neighboring cells [54]. It is interesting to note that one of the specimens in this study (Specimen # 1) in which the tumor grew to a larger size, occurred in an ear that had Meniere’s disease as well. Three other ears with CVS in this study had Otosclerosis (Specimen #s 7,8,9). Two of the smaller tumors occurred in individuals with evidence of other known previous inflammatory processes such as scarlet fever (Specimen #19) and chronic otitis media (Specimen #3). A third specimen (Specimen #8) also had an arachnoid cyst, which if typed as secondary could be the result of the tumor, infection, or trauma [57]. Additionally, Specimen #11 and #19 demonstrated fibrosis and osteoid formation, two common indicators of prior inflammation. Stressed and/or inflamed ears may be more susceptible to neoplasm.

Macrophages exist along the VIIIth nerve in the IAC and in the modiolus in the normal ear. Activation of macrophages is known to cause demyelination and remyelination in other peripheral disorders [58,59]. It is known that these repeated cycles of demyelination and remyelination are the cause of onion bulb formations within a nerve [35]. There were onion bulbs present in the tumors presented here. Many fibers appeared in an orientation that was orthogonal to their original plane. If Schwann cells lose contact with their axon, either through crush trauma or inflammation [60], they may repeatedly try to remyelinate a nerve. During this process, perhaps the possibility is greater for neoplastic transformation [61,62] of Schwann cells facing chronic irritation from demyelination and remyelination. It is not unreasonable to think about a scenario in which cochleovestibular schwannomas originate at a site of inflammation.

It is already known that inflammation contributes to the volume growth of established cochleovestibular schwannomas [49,50]. Nonsteroidal anti-inflammatory drugs such as aspirin can slow the growth of these tumors in vitro [63,64]. The mechanism is not completely understood but is likely through inhibition of COX-2 and prostaglandin E2 [65]. Work in mouse studies has shown that secretory factors from both good- and poor-hearing human schwannomas when applied to healthy mouse cochlear explants can result in nerve fiber disarray initially [20]. This finding was also true of tumorlets and tumors in the present study. Nerve fibers displayed a disorganized morphology. If fibers are disorganized from the initial stage of tumorlet formation, then it seems possible that fiber disorganization could be an initiative step in at least some of these tumors. Fiber disorganization continued to be a finding as tumors grew both in sporadic cases and in NF2. A mouse study [20] identified one of the secretory factors from the poor-hearing ears (with schwannomas) as TNFα. One of the largest producers of TNFα, an inflammatory cytokine, is macrophages [66]. Taken together, macrophages in the normal ear and macrophages in the smallest tumorlets, and macrophages in large tumors add to the evidence for an inflammatory component to the initiation and growth of cochleovestibular schwannomas. It was the father of modern pathology, Rudolf Virchow, who believed there was a relationship between chronic inflammation and cancer dating back to the 19th century [54,56].

In this study, tumorlets, as well as larger sporadic tumors, were at times filled with anti-neurofilament/and or anti-myelin protein zero labeling and demonstrated fibers taking tortuous routes. It may be noted that the smaller tumors often had more axons present than the larger tumors. In the 1980s, other researchers also found numerous nerve fibers within the body of cochleovestibular schwannomas [23]. Our results are consistent with findings that were published 30 years ago. Still other studies of surgical specimens found no fibers at all. One can imagine there are areas within all resected tumors that will harbor no remaining neurofilaments. There were areas, especially in the larger tumors in our study, devoid of neurofilament labeling. Presumably this is due to continued degeneration of the axons. A possible reason why our study found axons in cochleovestibular schwannomas while some studies on surgical specimens have not is most likely due to the thickness of our sample sections and the ability to survey the entire volume of these tumors. The power of this study was the ability to sample various levels of the entire volumes of these tumors as well as different-sized tumors. We were able to look at the most superior surface, the middle, and the inferior portions. Additionally, we could observe each tumor’s relation to other auditory and vestibular structures.

So, what do our findings reveal in terms of retro cochlear hearing loss? It appears from our study that degeneration of nerve fibers and myelin occurs in even the smallest tumors we were able to study. Damage to myelin along the nerve might cause changes in conduction velocities. Whether or not this would be perceived by the patient as a hearing or vestibular deficit is unknown, but demyelination associated with other disorders such as multiple sclerosis certainly results in associated hearing loss [67]. The early demyelination maybe manifest clinically with delayed or absent ABR and/or poor speech discrimination out of proportion to the loss of pure tone thresholds. Activated macrophages within the tumor or along the nerve could hasten demyelination and excrete toxic substances within the cochlea contributing to increased inflammation and perhaps worsening hearing loss as well.

In a recent study to identify FDA-approved drugs which might be repositioned for use against CVS, human CVS gene expression profiles were compared to databases of multi-drug exposure profiles or known gene–drug interactions. A calculated “connectivity score” was generated to match differential gene expression patterns characteristic of CVS with known interactions between FDA-approved drugs [68]. From 1155 FDA-approved drugs, 8 drugs with potential for repositioning appeared in both analysis of sporadic and NF2-associated VS. Four of the eight drugs identified were termed anti-inflammatory drugs, although anti-inflammatory drugs represented only 1.4% of the FDA-approved drugs screened. Perhaps this finding relates to the role of macrophages in sustaining the necessary microenvironment for VS growth. The anti-inflammatory effect of steroids to restore hearing in CVS patients, even if only temporarily, may indicate suppression of the inflammatory process and excretion of toxic substances to the cochlea [69,70]. Likewise, bevacizumab, although primarily identified as a vascular epithelial growth inhibitor, also has anti-inflammatory effects and can reduce hypoxia-driven inflammatory cytokine production. This may be a mechanism by which 35–40% of NF2-related schwannomatosis patients demonstrate hearing improvement [71,72]. Anti-VEGF therapies may, in part, work directly on macrophages [73]. Macrophages can play a pro-angiogenic role in the tumor microenvironment by secreting pro-angiogenic growth factors and facilitating degradation of extracellular matrix surrounding vessels [74]. Macrophages express VEGF [75,76,77] and can express VEGF-A when they are polarized toward an inflammatory phenotype [75,77]. So, anti-VEGF therapy can perhaps modulate inflammation within a tumor [73,78]. Macrophages can also express VEGFR-3 under certain conditions and then will respond to VEGF-C and VEGF-D [75]. These interactions can influence migration of immune cells and macrophage phenotype plasticity (from pro- to anti-inflammatory) [75,79].

Combined with the recent finding of abundant resident macrophages along the VIIIth nerve within the cochlea, and prevalent macrophages within these tumors, a new potential mechanism that sporadic cochleovestibular schwannomas may originate as a site of inflammation should be investigated further. Additionally, the presence of macrophages in these tumors provides another target for potential therapeutics. It is intriguing that the axonal disarray and early axonal degeneration present in these tumorlets together with the presence of macrophages. We feel further study is warranted.

This study is limited by its small sample size and modest availability of audiologic data. Causality cannot be established using histological tissue samples from human inner ears. Such tissues provide only a single time point in the progression of disease. The tissue samples utilized here have varied post-mortem times which can impact tissue staining. Tissue fixation, processing, embedding media [80], and storage times are potential confounding variables. It will be important to corroborate our findings in future studies of larger cohorts with well-documented clinical and histopathological data.

## 5. Conclusions

To summarize, one of the most important findings of this study appears to be the possibility that cochleovestibular schwannomas start as a spheroid of tangled fibers in disarray with macrophage involvement. Whether or not such a complex mass of axons constitutes a discrete tumor, meaning that the NF2 gene has been mutated, is beyond the scope of this paper. These tumorlets are identified as schwannomas by neuropathologists with conventional hematoxylin and eosin staining. What became evident in this study is the progression from small tumors seemingly filled with axons to larger tumors sometimes with very few axons remaining. This challenges prior theories on how schwannomas are thought to grow. Previously tumor cells in schwannomas were described not to infiltrate between nerve fibers but that they expand within a capsule and thereby compress the eighth nerve. More recently, other groups have reported that schwannomas may be growing in a fashion that more closely resembles neurofibroma growth. For example, the expression of macrophage colony stimulating factor (M-CSF) has been shown to be highly associated with aggressive VS growth [81]. Likewise, NF2-associated and sporadic VS were found to demonstrate a close association between vascularity and ionized calcium binding adapting molecule (Iba1+) macrophage density. Vascular endothelial growth factor (VEGF) was expressed by Iba1+ macrophages [76]. It has been long known that macrophage proliferation is essential to neurofibroma formation in neurofibromatosis type 1, and targeted therapy to reduce macrophage numbers resulted in decreased Schwann cell proliferation and increased Schwann cell death [82].

## Figures and Tables

**Table 1:** Specimen ID#, gender, age, and speech discrimination along with tumor characteristics.

ID #	Sex	Pattern of Involvement, Infiltrating (I)/ Pushing (*p*)	Tumor Type, Antoni A/ Antoni B	Tumor Size (mm) W × L × H	Occult/ Clinical	ABR	Speech Discrimination %, ear w/Tumor	Age at Death
1	F	I + *p*	A + B	4.6 × 9.4 × 5.4	Occult	NA	80	74
2	M	I + *p*	A + B	10.0 × 11.0 × 8.0	Clinical	Prolonged latencies waves I-III	32	75
3	F	I + *p*	A + B	1.0 × 2.1 × 2.8	Occult	NA	56	94
4	M	I + *p*	A + B	4.9 × 6.7 × 8.0	Occult	NA	NA	81
5	M	I + *p*	A + B	5.3 × 8.4 × 6.6	Occult	NA	NA	74
6	F	I + *p*	A + B	15.0 × 17.0 × 7.0	Clinical	NA	0	100
7	M	I + *p*	A + B	1.0 × 1.1 × 1.0	Occult	NA	48	71
8	F	I + *p*	A + B	0.5 × 0.9 × 1.0	Occult	NA	100	74
9	M	I + *p*	A + B	1.2 × 1.9 × 1.2	Occult	NA	56	84
10	M	I + *p*	A + B	1.6 × 1.6 × 2.4	Occult	NA	NA	49
11	F	I + *p*	A + B	2.5 diameter	Occult	NA	28	95
12	F	I + *p*	A + B	Extensive	Clinical	Retro cochlear indicated	56	43
13	M	I + *p*	A + B	Extensive	Clinical	NA	76	70
14	M	I + *p*	B	3.0 × 2.85 × 1.2	Occult	NA	NA	86
15	F	I + *p*	A + B	2.2 × 2.1 × 2.2	Occult	NA	NA	91
16	M	I	Mostly B	0.3 × 0.3 × 1.0	Occult	NA	NA	82
17	F	I	B	0.4 × 0.2 × 1.0	Occult	NA	NA	90
18	M	I + *p*	A + B	4.6 × 6.0 × 4.6	Occult	NA	NA	82
19	F	I + *p*	A + B	1.3 × 1.2 × 1.8	Occult	NA	NA	99
20	M	I + *p*	A + B	3.0 × 9.0	NA	NA	NA	44
21	M	I + *p*	A + B	6.5 × 14.0	NA	NA	NA	76
22	M	I + *p*	A + B	1.8 × 2.7	NA	NA	NA	67
23	M	I + *p*	A + B	1.8 × 5.6	NA	NA	NA	81
24	M	I + *p*	A + B	2.5 × 3.2	NA	NA	NA	68
25	M	I + *p*	A + B	5.0 × 4.0	NA	NA	NA	86
26	M	I + *p*	A + B	1.3 × 2.5	NA	NA	NA	77
27	M	I + *p*	A + B	2.6 × 5.2	NA	NA	NA	72
28	M	I + *p*	A + B	2.5 × 7.0	NA	NA	NA	72

There were two very small intralabyrinthine cochlear schwannomas (ID#16 and #17, Table 2). Of the remaining 26 tumors, 5 tumors originated within the cochlear nerve (Table 2). Although only 5 of the tumors originated from the cochlear portion of the VIIIth nerve, 21 of them invaded the cochlear nerve (checkmarks in Table 2). (NA means data Not Available).

**Table 3:** Positive immunolabeling for axons and myelin in CVS.

ID #	Slide #s with Positive Anti-NF Staining Within Tumor	Slide #s with Positive Anti-MPZ Staining Within Tumor	Slide #s with Evidence of Wallerian Degeneration Within Tumor
1	122, 230	119, 223, 229	122, 230
2	102, 270, 352	103, 269, 353	102, 270
3	190, 320, 360	192, 322, 362	190, 360
4	40, 141, 303	151, 300	141, 303
5	170, 228	171, 231, 312	170
6	16, 150, 280	17, 149, 282	16, 150
7	112, 131, 149	113, 132, 151	112, 131
8	262, 283, 300	263, 284, 302	262, 283, 300
9	89, 109, 132	113, 134	109, 132
10	212, 262, 312	263, 310	262, 312
11	152, 192, 239	153, 193, 240	152, 192
12	110, 240, 330	109, 239, 332	110, 240, 330
13	130, 221	132, 222	221
14	262	264	262
15	205	207	205
16	239, 249	240	249
17	205	204	205
18	72, 178, 242	73, 179, 243	178
19	179	182	179
20	78, 162, 242	169, 247	78, 162, 246
21	446, 553, 717	452, 558, 725	446, 553, 717
22	285, 313, 336	338	285, 313, 336
23	64, 126, 171	73, 181	64, 126, 171
24	377, 412, 452	380, 417, 456	377, 412, 452
25	453	328, 458, 526	
26	68, 117, 192	77, 128, 195	68, 117, 192
27	359, 444, 493	365, 455, 486	359, 444, 493
28	24, 127, 248	37, 132, 265	248

## Data Availability

The data that support the findings of this study are available from the corresponding author upon reasonable request.

## Supplementary Materials

The following supporting information can be downloaded at: https://www.mdpi.com/article/10.3390/biology14111540/s1, Figure S1: Study Workflow.

## Abbreviations

The following abbreviations are used in this manuscript:

ABR	Auditory Brainstem Response
NF	Neurofilament
MPZ	Myelin Protein Zero
CVS	Cochleovestibular Schwannomas
NF2	Neurofibromatosis Type 2
SNHL	Sensorineural Hearing Loss
IAC	Internal Auditory Canal
H&E	Hematoxylin and Eosin
Iba1	Ionized Calcium Binding Adaptor Molecule 1
ROS	Reactive Oxygen Species
VEGF	Vascular Endothelial Growth Factor
DAB	Diaminobenzidine

## References

[1] Roosli C., Linthicum F.H., Cureoglu S., Merchant S.N. (2012). What is the site of origin of cochleovestibular schwannomas?. Audiol. Neurootol..

[2] Gonzalez Revilla A. (1948). Differential diagnosis of tumors at the cerebellopontile recess. Bull. Johns Hopkins Hosp..

[3] Halliday J., Rutherford S.A., McCabe M.G., Evans D.G. (2018). An update on the diagnosis and treatment of vestibular schwannoma. Expert. Rev. Neurother..

[4] Carlson M.L., Smadbeck J.B., Link M.J., Klee E.W., Vasmatzis G., Schimmenti L.A. (2018). Next Generation Sequencing of Sporadic Vestibular Schwannoma: Necessity of Biallelic NF2 Inactivation and Implications of Accessory Non-NF2 Variants. Otol. Neurotol..

[5] Plotkin S.R., Messiaen L., Legius E., Pancza P., Avery R.A., Blakeley J.O., Babovic-Vuksanovic D., Ferner R., Fisher M.J., Friedman J.M. (2022). Updated diagnostic criteria and nomenclature for neurofibromatosis type 2 and schwannomatosis: An international consensus recommendation. Genet. Med..

[6] Gupta V.K., Thakker A., Gupta K.K. (2020). Vestibular Schwannoma: What We Know and Where We are Heading. Head Neck Pathol..

[7] Seizinger B.R., Martuza R.L., Gusella J.F. (1986). Loss of genes on chromosome 22 in tumorigenesis of human acoustic neuroma. Nature.

[8] Rouleau G.A., Seizinger B.R., Wertelecki W., Haines J.L., Superneau D.W., Martuza R.L., Gusella J.F. (1990). Flanking markers bracket the neurofibromatosis type 2 (NF2) gene on chromosome 22. Am. J. Hum. Genet..

[9] Welling D.B. (1998). Clinical manifestations of mutations in the neurofibromatosis type 2 gene in vestibular schwannomas (acoustic neuromas). Laryngoscope.

[10] Selters W.A., Brackmann D.E. (1977). Acoustic tumor detection with brain stem electric response audiometry. Arch. Otolaryngol..

[11] Eckermeier L., Pirsig W., Mueller D. (1979). Histopathology of 30 non-operated acoustic schwannomas. Arch. Otorhinolaryngol..

[12] Johnsson L.G., Hawkins J.E., Rouse R.C. (1984). Sensorineural and vascular changes in an ear with acoustic neurinoma. Am. J. Otolaryngol..

[13] Bozorg Grayeli A., Refass A., Smail M., Elgarem H., Kalamarides M., Bouccara D., Sterkers O. (2008). Diagnostic value of auditory brainstem responses in cerebellopontine angle tumours. Acta Otolaryngol..

[14] Al-Asad R.K., Montigny D.J., O’Malley J.T., Welling D.B., Jung D.H., Eckhard A.H., Kempfle J.S. (2025). Investigating cochlear cellular dynamics in neurofibromatosis type 2-associated schwannomatosis: A histopathological study. Front. Neurol..

[15] Nadol J.B., Diamond P.F., Thornton A.R. (1996). Correlation of hearing loss and radiologic dimensions of vestibular schwannomas (acoustic Neuromas). Am. J. Otol..

[16] Mahmud M.R., Khan A.M., Nadol J.B. (2003). Histopathology of the inner ear in unoperated acoustic neuroma. Ann. Otol. Rhinol. Laryngol..

[17] Merchant S.N., McKenna M.J., Merchant S.N., Nadol J.B. (2010). Neoplastic Growth. Schuknecht’s Pathology of the Ear.

[18] Roosli C., Linthicum F.H., Cureoglu S., Merchant S.N. (2012). Dysfunction of the cochlea contributing to hearing loss in acoustic neuromas: An underappreciated entity. Otol. Neurotol..

[19] Dilwali S., Lysaght A., Roberts D., Barker F.G., McKenna M.J., Stankovic K.M. (2013). Sporadic vestibular schwannomas associated with good hearing secrete higher levels of fibroblast growth factor 2 than those associated with poor hearing irrespective of tumor size. Otol. Neurotol..

[20] Dilwali S., Landegger L.D., Soares V.Y., Deschler D.G., Stankovic K.M. (2015). Secreted Factors from Human Vestibular Schwannomas Can Cause Cochlear Damage. Sci. Rep..

[21] Eggink M.C., Frijns J.H.M., Sagers J.E., O’Malley J.T., Liberman M.C., Stankovic K.M. (2022). Human vestibular schwannoma reduces density of auditory nerve fibers in the osseous spiral lamina. Hear. Res..

[22] Tesarova M., Peterkova L., Stastna M., Kolar M., Lacina L., Smetana K., Hynek R., Betka J., Vlasak A., Lukes P. (2023). Tumor Biology and Microenvironment of Vestibular Schwannoma-Relation to Tumor Growth and Hearing Loss. Biomedicines.

[23] Neely J.G. (1981). Gross and microscopic anatomy of the eighth cranial nerve in relationship to the solitary schwannoma. Laryngoscope.

[24] Feany M.B., Anthony D.C., Fletcher C.D. (1998). Nerve sheath tumours with hybrid features of neurofibroma and schwannoma: A conceptual challenge. Histopathology.

[25] Wippold F.J., Lubner M., Perrin R.J., Lammle M., Perry A. (2007). Neuropathology for the neuroradiologist: Antoni A and Antoni B tissue patterns. AJNR Am. J. Neuroradiol..

[26] Gompel V., Jamie J., Carlstrom L.P., Hadjipanayis C.G., Graffeo C.S., Patel N., Carlson M.L., Jacob J., Olson J.J. (2025). Congress of Neurological Surgeons Systematic Review and Evidence-Based Guideline on Surgical Resection for the Treatment of Patients With Vestibular Schwannomas: Update. Neurosurgery.

[27] Corfas G., Velardez M.O., Ko C.P., Ratner N., Peles E. (2004). Mechanisms and roles of axon-Schwann cell interactions. J. Neurosci..

[28] Wechsler J., Lantieri L., Zeller J., Voisin M.C., Martin-Garcia N., Wolkenstein P. (2003). Aberrant axon neurofilaments in schwannomas associated with phacomatoses. Virchows Arch..

[29] Neuroma A. (1991). Acoustic Neuroma. NIH Consens. Statement.

[30] Hung G., Colton J., Fisher L., Oppenheimer M., Faudoa R., Slattery W., Linthicum F. (2002). Immunohistochemistry study of human vestibular nerve schwannoma differentiation. Glia.

[31] Ylikoski J., Palva T., Collan Y. (1978). Eighth nerve in acoustic neuromas. Special reference to superior vestibular nerve function and histopathology. Arch. Otolaryngol..

[32] Ylikoski J., Collan Y., Palva T., Jauhiainen T. (1978). Cochlear nerve in neurilemomas. Audiology and histopathology. Arch. Otolaryngol..

[33] Neely J.G., Armstrong D., Benson J., Neblett C. (1981). “Onion bulb” formation associated with a solitary neoplasm of the eighth nerve sheath. Am. J. Otolaryngol..

[34] Neely J.G., Hough J. (1986). Histologic findings in two very small intracanalicular solitary schwannomas of the eighth nerve. Ann. Otol. Rhinol. Laryngol..

[35] Neely J.G., Hough J.V. (1988). Histologic findings in two very small intracanalicular solitary schwannomas of the eighth nerve: II. “Onion bulbs”. Am. J. Otol..

[36] Forton G., Moeneclaey L., Declau F., Marquet J. (1989). The involvement of the cochlear nerve in neurinomas of the eighth cranial nerve. Arch. Otorhinolaryngol..

[37] Marquet J.F., Forton G.E., Offeciers F.E., Moeneclaey L.L. (1990). The solitary schwannoma of the eighth cranial nerve. An immunohistochemical study of the cochlear nerve-tumor interface. Arch. Otolaryngol. Head Neck Surg..

[38] Hannan C.J., Raghunathan A., Van Gompel J.J., Pathmanaban O. (2025). Pathology and tumor microenvironment of vestibular schwannoma. Handb. Clin. Neurol..

[39] Nickl V., Ziebolz D., Rumpel C., Klein D., Nickl R., Rampeltshammer E., Monoranu C.M., Ernestus R.I., Matthies C., Lohr M. (2024). Analysis of tumor microenvironment composition in vestibular schwannomas: Insights into NF2-associated and sporadic variations and their clinical correlations. Front. Oncol..

[40] Nguyen H.T.N., Duhon B.H., Kuo H.C., Fisher M., Brickey O.M., Zhang L., Otero J.J., Prevedello D.M., Adunka O.F., Ren Y. (2024). Matrix metalloproteinase 9: An emerging biomarker for classification of adherent vestibular schwannoma. Neurooncol. Adv..

[41] Duhon B.H., Thompson K., Fisher M., Kaul V.F., Nguyen H.T., Harris M.S., Varadarajan V., Adunka O.F., Prevedello D.M., Kolipaka A. (2024). Tumor biomechanical stiffness by magnetic resonance elastography predicts surgical outcomes and identifies biomarkers in vestibular schwannoma and meningioma. Sci. Rep..

[42] O’Malley J.T., Burgess B.J., Jones D.D., Adams J.C., Merchant S.N. (2009). Techniques of celloidin removal from temporal bone sections. Ann. Otol. Rhinol. Laryngol..

[43] O’Malley J.T., Nadol J.B., McKenna M.J. (2016). Anti CD163+, Iba1+, and CD68+ Cells in the Adult Human Inner Ear: Normal Distribution of an Unappreciated Class of Macrophages/Microglia and Implications for Inflammatory Otopathology in Humans. Otol. Neurotol..

[44] Escalona-Zapata J., Diez Nau M.D. (1978). The nature of macrophages (foam cells) in neurinomas. Tissue culture study. Acta Neuropathol..

[45] Rossi M.L., Jones N.R., Esiri M.M., Havas L., Nakamura N., Coakham H.B. (1990). Mononuclear cell infiltrate, HLA-Dr expression and proliferation in 37 acoustic schwannomas. Histol. Histopathol..

[46] Gomez-Brouchet A., Delisle M.B., Cognard C., Bonafe A., Charlet J.P., Deguine O., Fraysse B. (2001). Vestibular schwannomas: Correlations between magnetic resonance imaging and histopathologic appearance. Otol. Neurotol..

[47] Park C.K., Kim D.C., Park S.H., Kim J.E., Paek S.H., Kim D.G., Jung H.W. (2006). Microhemorrhage, a possible mechanism for cyst formation in vestibular schwannomas. J. Neurosurg..

[48] Yokoo H., Oishi T., Isoda K., Nakazato Y., Toyokuni S. (2007). Oxidative stress is related to the formation of Antoni B patterns and eosinophilic hyaline droplets in schwannomas. Neuropathology.

[49] de Vries M., Hogendoorn P.C., Briaire-de Bruyn I., Malessy M.J., van der Mey A.G. (2012). Intratumoral hemorrhage, vessel density, and the inflammatory reaction contribute to volume increase of sporadic vestibular schwannomas. Virchows Arch..

[50] de Vries M., Briaire-de Bruijn I., Malessy M.J., de Bruine S.F., van der Mey A.G., Hogendoorn P.C. (2013). Tumor-associated macrophages are related to volumetric growth of vestibular schwannomas. Otol. Neurotol..

[51] Coussens L.M., Werb Z. (2002). Inflammation and cancer. Nature.

[52] Jabaly S., Alhareth F., Jabaly N., Abboud F., Haddad S. (2025). Sciatic schwannoma allegedly following repeated trauma: A case report and exploration of a potential association. Int. J. Surg. Case Rep..

[53] Judd T., Jones T., Thornberry L. (2014). Schwannoma of the posterior tibial nerve: Case study. J. Am. Podiatr. Med. Assoc..

[54] Grivennikov S.I., Greten F.R., Karin M. (2010). Immunity, inflammation, and cancer. Cell.

[55] Schneider A.B., Ron E., Lubin J., Stovall M., Shore-Freedman E., Tolentino J., Collins B.J. (2008). Acoustic neuromas following childhood radiation treatment for benign conditions of the head and neck. Neuro Oncol..

[56] Kalisperati P., Spanou E., Pateras I.S., Korkolopoulou P., Varvarigou A., Karavokyros I., Gorgoulis V.G., Vlachoyiannopoulos P.G., Sougioultzis S. (2017). Inflammation, DNA Damage, Helicobacter pylori and Gastric Tumorigenesis. Front. Genet..

[57] Öcal E. (2023). Understanding intracranial arachnoid cysts: A review of etiology, pathogenesis, and epidemiology. Child’s Nerv. Syst..

[58] Davies C.L., Miron V.E. (2018). Distinct origins, gene expression and function of microglia and monocyte-derived macrophages in CNS myelin injury and regeneration. Clin. Immunol..

[59] Lund H., Pieber M., Parsa R., Grommisch D., Ewing E., Kular L., Han J., Zhu K., Nijssen J., Hedlund E. (2018). Fatal demyelinating disease is induced by monocyte-derived macrophages in the absence of TGF-beta signaling. Nat. Immunol..

[60] Belanger E., Henry F.P., Vallee R., Randolph M.A., Kochevar I.E., Winograd J.M., Lin C.P., Cote D. (2011). In vivo evaluation of demyelination and remyelination in a nerve crush injury model. Biomed. Opt. Express.

[61] Hannan C.J., Lewis D., O’Leary C., Donofrio C.A., Evans D.G., Roncaroli F., Brough D., King A.T., Coope D., Pathmanaban O.N. (2020). The inflammatory microenvironment in vestibular schwannoma. Neurooncol. Adv..

[62] Hannan C.J., Lewis D., O’Leary C., Donofrio C.A., Evans D.G., Stapleton E., Freeman S.R., Lloyd S.K., Rutherford S.A., Hammerbeck-Ward C. (2022). Beyond Antoni: A Surgeon’s Guide to the Vestibular Schwannoma Microenvironment. J. Neurol. Surg. B Skull Base.

[63] Kandathil C.K., Cunnane M.E., McKenna M.J., Curtin H.D., Stankovic K.M. (2016). Correlation Between Aspirin Intake and Reduced Growth of Human Vestibular Schwannoma: Volumetric Analysis. Otol. Neurotol..

[64] Kandathil C.K., Dilwali S., Wu C.C., Ibrahimov M., McKenna M.J., Lee H., Stankovic K.M. (2014). Aspirin intake correlates with halted growth of sporadic vestibular schwannoma in vivo. Otol. Neurotol..

[65] Dilwali S., Kao S.Y., Fujita T., Landegger L.D., Stankovic K.M. (2015). Nonsteroidal anti-inflammatory medications are cytostatic against human vestibular schwannomas. Transl. Res..

[66] Parameswaran N., Patial S. (2010). Tumor necrosis factor-alpha signaling in macrophages. Crit. Rev. Eukaryot. Gene Expr..

[67] Di Stadio A., Dipietro L., Ralli M., Meneghello F., Minni A., Greco A., Stabile M.R., Bernitsas E. (2018). Sudden hearing loss as an early detector of multiple sclerosis: A systematic review. Eur. Rev. Med. Pharmacol. Sci..

[68] Sagers J.E., Brown A.S., Vasilijic S., Lewis R.M., Sahin M.I., Landegger L.D., Perlis R.H., Kohane I.S., Welling D.B., Patel C.J. (2018). Computational repositioning and preclinical validation of mifepristone for human vestibular schwannoma. Sci. Rep..

[69] Huynh P.P., Saba E.S., Hoerter J.E., Jiang N. (2023). Steroid Efficacy on Audiologic Recovery in Patients With Sudden Sensorineural Hearing Loss and Vestibular Schwannoma: A Retrospective Review. Otol. Neurotol..

[70] Puccinelli C., Carlson M.L. (2019). Improvement or Recovery From Sudden Sensorineural Hearing Loss With Steroid Therapy Does Not Preclude the Need for MRI to Rule Out Vestibular Schwannoma. Otol. Neurotol..

[71] Plotkin S.R., Allen J., Dhall G., Campian J.L., Clapp D.W., Fisher M.J., Jain R.K., Tonsgard J., Ullrich N.J., Thomas C. (2023). Multicenter, prospective, phase II study of maintenance bevacizumab for children and adults with NF2-related schwannomatosis and progressive vestibular schwannoma. Neuro Oncol..

[72] Ribatti D. (2022). Immunosuppressive effects of vascular endothelial growth factor. Oncol. Lett..

[73] Gregory G.E., Haley M.J., Jones A.P., Hannan C.J., Evans D.G., King A.T., Paszek P., Pathmanaban O.N., Couper K.N., Brough D. (2024). Alternatively activated macrophages are associated with faster growth rate in vestibular schwannoma. Brain Commun..

[74] De Palma M., Biziato D., Petrova T.V. (2017). Microenvironmental regulation of tumour angiogenesis. Nat. Rev. Cancer.

[75] Kannan S., Rutkowski J.M. (2024). VEGFR-3 signaling in macrophages: Friend or foe in disease?. Front. Immunol..

[76] Lewis D., Donofrio C.A., O’Leary C., Li K.L., Zhu X., Williams R., Djoukhadar I., Agushi E., Hannan C.J., Stapleton E. (2021). The microenvironment in sporadic and neurofibromatosis type II-related vestibular schwannoma: The same tumor or different? A comparative imaging and neuropathology study. J. Neurosurg..

[77] Li Y.L., Zhao H., Ren X.B. (2016). Relationship of VEGF/VEGFR with immune and cancer cells: Staggering or forward?. Cancer Biol. Med..

[78] Motz G.T., Coukos G. (2011). The parallel lives of angiogenesis and immunosuppression: Cancer and other tales. Nat. Rev. Immunol..

[79] Cursiefen C., Chen L., Borges L.P., Jackson D., Cao J., Radziejewski C., D’Amore P.A., Dana M.R., Wiegand S.J., Streilein J.W. (2004). VEGF-A stimulates lymphangiogenesis and hemangiogenesis in inflammatory neovascularization via macrophage recruitment. J. Clin. Investig..

[80] O’Malley J.T., Merchant S.N., Burgess B.J., Jones D.D., Adams J.C. (2009). Effects of fixative and embedding medium on morphology and immunostaining of the cochlea. Audiol. Neurootol..

[81] de Vries W.M., Briaire-de Bruijn I.H., van Benthem P.P.G., van der Mey A.G.L., Hogendoorn P.C.W. (2019). M-CSF and IL-34 expression as indicators for growth in sporadic vestibular schwannoma. Virchows Arch..

[82] Fletcher J.S., Springer M.G., Choi K., Jousma E., Rizvi T.A., Dombi E., Kim M.O., Wu J., Ratner N. (2019). STAT3 inhibition reduces macrophage number and tumor growth in neurofibroma. Oncogene.

