# Supplementary figures and images for: Immunohistopathology of Cochleovestibular Schwannoma in Human Temporal Bone Specimens
Source: Biology (Basel). 2025 Nov 3;14(11):1540. doi: 10.3390/biology14111540 (PMC12650569; doi:10.3390/biology14111540)

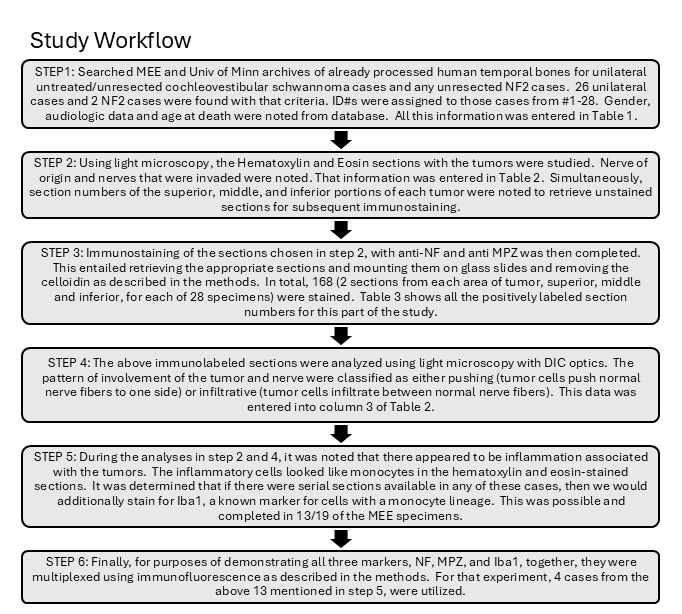

Supplement: Supplementary file 1 [file biology-14-01540-s001.zip › biology-3881770-supplementary.tif]
